# Effect of butorphanol on visceral pain in patients undergoing gastrointestinal endoscopy: a randomized controlled trial

**DOI:** 10.1186/s12871-023-02053-9

**Published:** 2023-03-28

**Authors:** Jing Wang, Xinyan Wang, Haiyang Liu, Ruquan Han

**Affiliations:** 1 Department of Anesthesiology, Beijing Tiantan Hospital, Capital Medical University, No. 119, Southwest 4Th Ring RD, Fengtai District, Beijing, 100070 PR China; 2 Department of Anesthesiology, DaxingDistrict, Beijing Daxing People’s Hospital, No. 26, Huangcun West Street, Beijing, People’s Republic of China

**Keywords:** Butorphanol, Visceral pain, Propofol, Gastroscopy, Colonoscopy

## Abstract

**Background:**

Butorphanol slightly influences the respiratory and circulatory systems, has a better effect on relieving the discomfort caused by mechanical traction, and has a low incidence of postoperative nausea and vomiting (PONV). Combined butorphanol and propofol may suppress postoperative visceral pain, which is avoidable in gastrointestinal endoscopy. Thus, we hypothesized that butorphanol could decrease the incidence of postoperative visceral pain in patients undergoing gastroscopy and colonoscopy.

**Methods:**

This was a randomized, placebo-controlled, and double-blinded trial. Patients undergoing gastrointestinal endoscopy were randomized to intravenously receive either butorphanol (Group I) or normal saline (Group II). The primary outcome was visceral pain after the procedure 10 min after recovery. The secondary outcomes included the rate of safety outcomes and adverse events. Postoperative visceral pain was defined as a visual analog scale (VAS) score ≥ 1.

**Results:**

A total of 206 patients were enrolled in the trial. Ultimately, 203 patients were randomly assigned to Group I (*n* = 102) or Group II (*n* = 101). In total, 194 patients were included in the analysis: 95 in Group I and 99 in Group II. The incidence of visceral pain at 10 min after recovery was found to be statistically lower with butorphanol than with the placebo (31.5% *vs*. 68.5%, respectively; RR: 2.738, 95% CI [1.409–5.319], *P* = 0.002), and the notable difference was in pain level or distribution of visceral pain (*P* = 0.006).

**Conclusions:**

The trial indicated that adding butorphanol to propofol results in a lower incidence of visceral pain after surgery without noticeable fluctuations in circulatory and respiratory functions for gastrointestinal endoscopy patients.

**Trial registration:**

Clinicaltrials.gov NCT04477733 (PI: Ruquan Han; date of registration: 20/07/2020).

**Supplementary Information:**

The online version contains supplementary material available at 10.1186/s12871-023-02053-9.

## Background

More than two million people die of gastrointestinal cancer, accounting for approximately 60% of new cases, based on Global Cancer Statistics 2018 [1]. Gastrointestinal endoscopy is the most popular measure to screen and diagnose gastrointestinal cancer [2]. A recent study showed that the incidence of moderate or severe abdominal pain after colonoscopy was 16.7% (601 of 3611) in participants examined with standard air insufflation [3]. More people prefer painless endoscopy for more comfortable medical experiences and patient safety.

Propofol is widely used in endoscopy due to its fast onset of action, short action time, and quick and complete postoperative recovery [4–6]. However, the use of propofol alone varies, and increasing the dose causes circulatory and respiratory depression related to the infusion dose and rate [6, 7]. Therefore, the combination of propofol and low-dose opioids has been promoted to reduce propofol consumption and adverse effects [8, 9].

Butorphanol is a mixed opioid receptor agonist and antagonist that acts on κ receptors [10]. It has a faster onset through intravenous injection, a lower risk of addiction with a single dose, and a more prolonged analgesic effect [11]. Butorphanol also slightly influences the respiratory and circulatory systems, has a better impact on relieving the discomfort caused by mechanical traction, and has a low incidence of postoperative nausea and vomiting (PONV) [11–14]. These advantages make it more suitable for painless gastrointestinal endoscopy. However, sedation can also cause dizziness, drowsiness, and other adverse reactions during recovery [15].

In this randomized controlled study, we intended to confirm that butorphanol can significantly improve postoperative visceral pain, reduce the dosage of propofol needed and improve quality in patients undergoing gastroscopy and colonoscopy.

## Methods

### Study design and participants

This dual-center, randomized, placebo-controlled study was conducted at Beijing Tiantan Hospital, Capital Medical University, and Beijing Daxing People’s Hospital between August 14th, 2020, and September 30th, 2021. This study was approved by the China Ethics Committee of Registering Clinical Trials (Registration number: ChiECRCT20200200) and registered at Clinicaltrials.gov (NCT04477733, 20/07/2020)) in July 2020. All patients or their legal representatives provided written informed consent. The study followed the Consolidated Standards of Reporting Trials (CONSORT) guidelines.

We recruited adult patients aged 18 to 65 with an American Society of Anesthesiologists physical status from I to III who underwent colonoscopy or gastroscopy. The exclusion criteria included patients with a body mass index > 30 kg/m^2^, a history of depression, opioid dependence, poorly controlled hypertension (systolic blood pressure > 180 mmHg), myocardial infarction, severe liver disease, and significant abdominal pain before surgery; patients with sensory system or language dysfunctions who could not cooperate to complete the scale; and pregnant women.

### Blinding and randomization

Patients were randomly assigned to receive butorphanol (Group I) or normal saline (Group II) in a 1:1 ratio based on computer-generated stratified randomization numbers. The random numbers were sealed in separate opaque envelopes until the analysis was complete. Patients in Group I received 10 μg/kg butorphanol intravenously 3 min before the intravenous injection of propofol. The patients assigned to Group II received an identical volume of normal saline at the same infusion rate.

The investigators did not participate in other processes while preparing the research solution. A designated staff prepared the solution, enclosed in a dark syringe (volume: 5 ml) labeled "study solution," The two solutions seemed identical. Randomization was blinded to the participants, chief anesthesiologists, and outcome assessors.

### Perioperative management

The regimens were standardized in both groups. Patients were deprived of water for 2 h and fasted for 8 h before surgery. Based on the requirements of anesthesia and surgery, venous access (central vein of the upper limb) was established. As perioperative fasting and bowel preparation are believed to cause intravascular hypovolemia, preemptive intravenous fluid was infused with 10–15 ml/kg normal saline. Standard intraoperative monitoring, which included electrocardiogram (ECG), heart rate (HR), blood pressure (BP), respiratory rate (RR), and pulse oxygen saturation (SpO_2_), was applied. Preinduction medication was not administered. The patient received preoxygenation with a mask filled with 100% oxygen (4–6 L/min) for at least 3–5 min before induction. The induction of anesthesia was performed with 1.5–2 mg/kg propofol as a bolus infused slowly (60 to 120 s) until the eyelash reflex disappeared. When the vital signs were stable, an endoscopic examination was started. If there was a body movement reaction during the examination, a dose of 0.5–1 mg/kg propofol was added. Moreover, the mean arterial pressure (MAP) and HR were maintained within ± 20% of the values before anesthesia induction during the procedure with vasoactive drugs such as dopamine and atropine. After the procedure, the patients were transferred to the postanesthetic care unit (PACU) and followed up regularly for any adverse events for at least 30 min. If the pain persisted after 30 min, regardless of the patient’s assigned group (Group I or Group II), a single dose (0.01 μg/kg) of sufentanil was given, and the patients could leave the PACU after the pain was relieved.

### Data collection and outcomes

Baseline data were recorded, such as age, sex, height, weight, body mass index, ASA physical status, and type of operation. The MAP, HR, RR, and SpO_2_ of the patients were recorded at seven time points: T0, at admission; T1, before anesthesia; T2, 5 min after propofol administration; T3, at the end of the operation; T4, after 5 min in the PACU; T5, after 10 min in the PACU; and T6, after 30 min in the PACU.

The primary outcome was the incidence of visceral pain after the procedure. Because there is no universal scale for quantifying visceral pain, we defined a visual analog scale (VAS) score of ≥ 1 10 min after recovery as visceral pain. The secondary outcomes were visceral pain at 20 and 30 min after recovery, propofol consumption, the incidence of injection pain caused by propofol, episodes of hypotension (defined as an MAP less than 60 mmHg or 30% of the values before the induction of anesthesia) or bradycardia (defined as an HR less than 60 bpm), the operation time, the recovery time, and the adverse events at 24 h after recovery, such as fatigue, nausea or vomiting, abdominal bloating, dizziness or headache, hypoxemia (blood oxygen saturation below 90% for more than a minute or requiring any airway intervention), and involuntary body movement.

### Sample size calculation

We used PASS software to calculate the necessary sample size for this study. A cohort study reported that 45% of participants (124 of 277) undergoing colonoscopy and gastroscopy complained of pain during follow-up [16]. The incidence of pain after colonoscopy was higher in two other randomized controlled studies, at 47.8% and 51%, respectively [17, 18]. In our pretrial test, the incidence of visceral pain after colonoscopy or gastroscopy was 54%. Combining all the above data, we estimated that the incidence of pain in Group II was 50%, with a 20% reduction in Group I. Thus, 186 people were included in this trial (power = 80%, and α = 0.05). The ratio of the two sets of samples was 1:1. Considering an overall withdrawal rate of 10%, the sample size was estimated to be 206 patients (103 patients per group).

### Statistical analysis

The data were analyzed using SPSS 26.0, and the figures were created by GraphPad Prism 9.0. All analyses were based on the intention-to-treat (ITT) principle. The Kolmogorov–Smirnov test was used to analyze continuous outcomes to judge the normality of their distributions. Normally distributed continuous variables were summarized as the mean value ± standard deviation and were compared using independent t tests. Skewed continuous variables were summarized as the median value and interquartile range and were compared using the Mann–Whitney U test. As appropriate, categorical variables were summarized as the number and percentage and compared using the chi-square or Fisher’s exact test. The primary endpoint was the incidence of visceral pain in the recovery room, and the chi-square test was used to compare the differences between the two groups. The risk ratios and 95% confidence intervals were reported for the primary and secondary outcomes. MAP, HR, RR, and SpO_2_ were compared four times between the groups at T0, T1, T2, and T3 using a two-sample Student’s t test. Bonferroni correction was used to justify the *P* values for these three variables, and an α level of 0.0125 was considered statistically significant. Moreover, an α level of 0.05 was considered statistically significant for the remaining variables.

## Results

### Patient demographics and perioperative characteristics

**Fig. 1:**
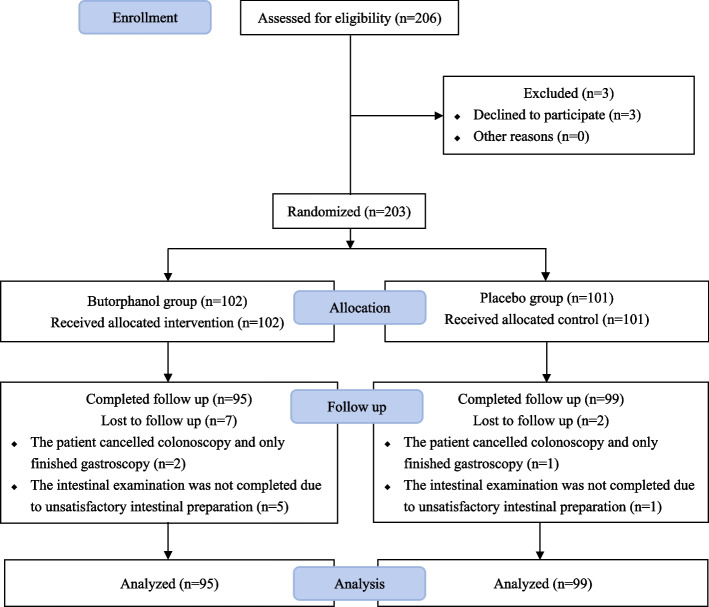
CONSORT flow diagram. CONSORT: Consolidated Standards of Reporting Trials

In the ITT population, 206 patients were consecutively enrolled at two hospitals between August 14th, 2020, and September 30th, 2021. Eventually, 203 patients were randomly assigned to Group I (*n* = 102) or Group II (*n* = 101). At the primary time point, nine patients were lost to follow-up. A total of 194 patients in the two groups completed the study (Fig. 1). The data from all 194 patients were included in the analysis. The demographics, baseline assessment, and intraoperative details were similar between the two groups and are presented in Table 1.

**Table 1:** Demographic and clinical features (median and interquartile range, mean and standard deviation, or number and percentage)

Characteristics	Group I: Butorphanol (*n* = 95)	Group II: Normal Saline (*n*-99)	Sig
Age(years)	49(23–67)	49(26–65)	0.727
Gender(male/female)	43/53	50/49	0.465
Height(cm)	167(9)	166(8)	0.898
Weight(kg)	68(12)	66(12)	0.248
BMI(kg/m^2^)	24.5(3.3)	24.8(3.0)	0.109
Smoking history(%)	26(27.4)	34(34.3)	0.293
Drinking history(%)	38(40.0)	43(43.4)	0.628
Hypertension(%)	31(32.6)	20(20.2)	0.049
Diabetes mellitus(%)	9(9.5)	7(7.1)	0.543
Hyperlipemia(%)	20(21.1)	17(17.2)	0.492
Heart disorders(%)	3(3.2)	5(5.1)	0.508
Coronary heart disease	1(1.1)	2(2.0)	
Heart valve disease	1(1.0)	0(0)	
Arrhythmia	0(0)	2(2.0)	
ASA physical status			
I	67	66	0.563
II	28	33	
Type of operations			
Total	95	99	0.886
Colonoscopy	47	48	
Gastro-colonoscopy	50	49	

*BMI* body mass index, *ASA* American Society of Anesthesiologists

### Visceral pain

**Fig. 2:**
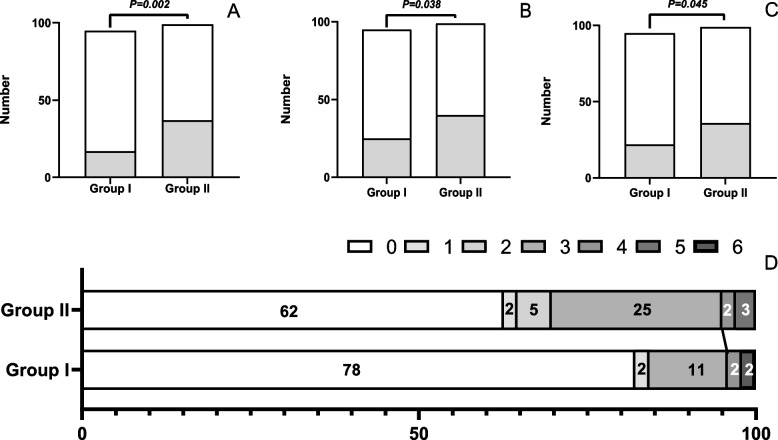
Incidence of visceral pain recovery at 10 (**A**), 20 (**B**), and 30 min (**C**) and distribution of visceral pain scores at 10 min (**D**). Data are *frequency.* Group I: Propofol and Butorphanol; Group II: Propofol and Saline. Visceral pain was defined as ≥ 1 of the visual analog score (VAS). The incidence of visceral pain at 10 min in the recovery room was found to be significantly lower in Group I than in Group II (propofol and butorphanol *vs.* propofol and saline, respectively; *χ2* = 9.157, *P* = 0.002, RR: 2.738, 95% CI [1.409- 5.319]. The white bar indicates patients with VAS 0, and the black bar indicates patients with more than VAS 1

The incidence of visceral pain at 10 min after recovery was significantly lower with butorphanol (31.5% *vs.* 68.5%, respectively; RR: 2.738, 95% CI [1.409–5.319], *χ2* = 9.157, *P* = 0.002; see Table 2). The significant difference in Fig. 2 and Table 2 shows the pain level and distribution of visceral pain (*P* = 0.006).

**Table 2:** Comparison of efficacy and safety outcomes (median and interquartile range or frequency and percentage)

	Group I: Propofol and Butorphanol (*n* = 95)	Group II: Propofol and Saline (*n*-99)	RR (95%CI)	*P*
Primary outcome(%)				
Visceral pain at 10 min after recovery	17 (31.5)	37(68.5)	2.738(1.409–5.319)	0.002
Pain level at 10 min after recovery(%)			NA	0.006
No pain (0)	78(82.1)	62(62.6)		
mild pain (1–3)	13(13.7)	32(32.3)		
moderate pain (4–6)	4(4.2)	5(5.1)		
severe pain (7–10)	0(0)	0(0)		
Secondary outcome				
Visceral pain at 20 min after recovery	25(38.5)	40(61.5)	1.898(1.033–3.487)	0.038
Visceral pain at 30 min after recovery	22(37.9)	36 (62.1)	1.896(1.011–3.555)	0.045
Propofol consumption (mg)	200(170–280)	250(200–320)	NA	0.007
Hypotension (%)	48(50.5)	44(44.4)	0.783(0.445- 1.378)	0.396
Bradycardia (%)	1(1.1)	0(0)	0.487(0.421- 0.563)	0.984
Incidence of injection pain (%)	7(7.4)	12(12.1)	1.734(0.652- 4.611)	0.266
Operation time(min)	15(12–21)	16(10–25)	NA	0.331
Recovery time (min)	25(18–30)	30(19–30)	NA	0.417

The incidence of visceral pain at 20 and 30 min after recovery showed a similar change (Fig. 2, Table 2). Butorphanol infusion allowed for an apparent reduction in propofol consumption (200 *vs.* 250, respectively; Z = -2.720, *P* = 0.007), which was more pronounced in patients undergoing colonoscopy than in those undergoing gastro-colonoscopy (Z = -6.999, *P* < 0.001; Online Resource 1). This may be because of the prolonged operation time in gastro-colonoscopy (Z = -3.095, *P* = 0.002; Online Resource 2). However, there were no significant differences in episodes of bradycardia or hypotension, operation time, recovery time, or incidence of injection pain between the two groups (*P* > 0.05, Table 2).

Perioperative monitoring parameters (MAP, SpO2 and RR).

**Fig. 3:**
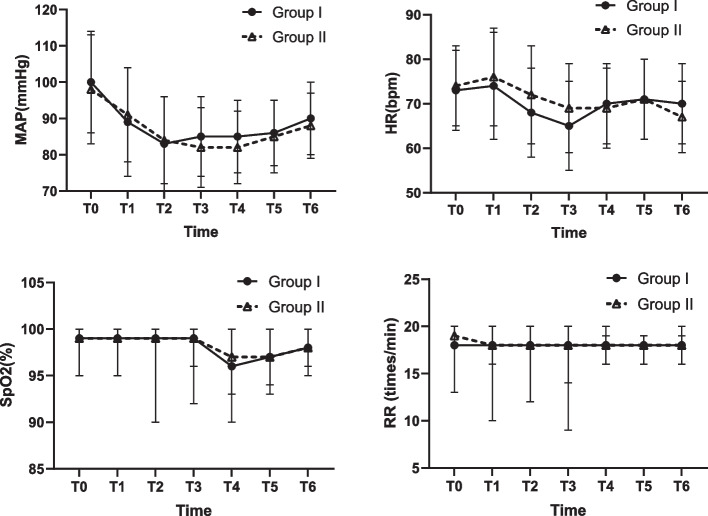
Changes in MAP, HR, SpO2, and RR. MAP, HR: Normal distribution, mean, and SD. SpO2, RR: Nonnormal distribution, median, and upper/lower limit. T0, at admission; T1, before anesthesia; T2, 5 min after propofol administration; T3, at the end of the operation; T4, 5 min in the PACU; T5, 10 min after recovery; T6, 30 min after recovery. Only after the administration (T2, t = -2.716, *P* = 0.007) and the end of the operation (T3, t = -2.261, *P* = 0.025) was the HR of Group I slightly lower than that of Group II, and the other time points were not significantly different

The MAP, SpO_2,_ and RR were not significantly different between the two groups of patients at the seven time points. Only after 5 min of propofol administration (T2, t = -2.716, *P* = 0.007) and at the end of the operation (T3, t = -2.261, *P* = 0.025) was the HR of Group I slightly lower than that of Group II, and the other time points were significantly different (Fig. 3).

Adverse events.

Only one patient in the butorphanol group developed fatigue during the operation and recovery room. After 24 h of recovery in the butorphanol group, the number of people with nausea or vomiting, abdominal bloating, dizziness, or headache was 2, 7, and 5, respectively. In Group II, only seven people had abdominal pain and bloating. No cases of hypoxemia or body movements were reported. There were no significant differences between the two groups in adverse events (*χ*^*2*^ = 4.345, *P* = 0.182).

## Discussion

This study demonstrates that butorphanol results in a statistically lower incidence of visceral pain after surgery and reduced propofol consumption for gastrointestinal endoscopy without noticeable fluctuations in circulatory and respiratory functions. Intravenous butorphanol neither prolongs recovery time nor increases adverse events.

First, we selected 10 μg/kg butorphanol as the administration dosage in this study. One reason is that the patients had minimal discomfort at this dose in our pretest. The other is that 9.07 μg/kg of butorphanol was more effective than sufentanil for gastrointestinal endoscopy sedation and notably reduced the recovery time [19]. However, Lv Sun and colleagues found that compared with other dosages of butorphanol (2.5, 5, or 10 μg/kg), intravenous preinjection of 7.5 μg/kg of butorphanol with propofol had the lowest incidence of body movement, drowsiness, and dizziness [20]. A 7.5 μg/kg dosage of butorphanol can be optimal for gastroscopy and colonoscopy patients. The difference may come from the sample size and clinicians.

The incidence of visceral pain was significantly lower in the butorphanol group at 10, 20, and 30 min after recovery. This is consistent with most previous studies [21, 22].

Butorphanol infusion allowed for a noticeable reduction in propofol consumption, which was more pronounced in colonoscopy than in gastro-colonoscopy. This may be because of the prolonged operation time in gastro-colonoscopy. Forster and colleagues found similar outcomes for lidocaine: lidocaine resulted in a 50% reduction in propofol dose requirements during colonoscopy and significantly lower postcolonoscopy pain and fatigue [23]. Propofol-based combination therapy reduces propofol consumption, resulting in fewer cardiopulmonary complications and improving anesthesia safety [23]. However, the incidence of propofol-related injection pain was not significantly different, which is inconsistent with previous studies [20, 24]. We may have selected a higher drug dose, or the sample size needed to be increased.

The incidence of hypoxemia was between 8.2% and 15% [20, 25], but we did not observe any cases of hypoxemia. The incidence of other adverse events was extremely low. Only one patient had fatigue; two patients had nausea or vomiting; fourteen had abdominal pain or bloating; and five had dizziness or headache. This was mainly because of our strict monitoring and perfect operation perioperative management in the clinical process. At the same time, there was no difference in the operation time, recovery time, or incidence of injection pain between the groups in our study. There was no clear distinction between the MAP and SpO_2_. RR or HR was found during the procedure. All these reasons prove the safety of butorphanol.

Our study should be interpreted with several limitations. As mentioned earlier, the high dosage of butorphanol (10 μg/kg) makes it difficult to find significant differences in the incidence of complications. We should conduct more research on the dosage of butorphanol. Finally, we recorded the total propofol consumption, so it is unclear which stage (the induction of sedation or during the infusion of study medications) caused the increase in the propofol dosage.

In conclusion, butorphanol decreases the incidence of visceral pain and propofol consumption for gastrointestinal endoscopy with minimal fluctuations in circulatory and respiratory functions. However, the rate of adverse events and recovery time did not differ significantly after the use of butorphanol. The clinical application needs to be weighed according to the actual situation of the patients.

## Supplementary Information

**Additional file 1.**

## Data Availability

The datasets generated and analyzed during the current study are not publicly available due to institutional restrictions but are available from the corresponding author on reasonable request.

## Abbreviations

PONV: Postoperative nausea and vomiting
VAS: Visual analog score
ECG: Electrocardiogram
HR: Heart rate
BP: Blood pressure
RR: Respiratory rate
SpO2: Pulse oxygen saturation
MAP: Mean arterial pressure
PACU: Postanesthetic care unit
ITT: Intention to treat
