# Supplementary material for: Effect of butorphanol on visceral pain in patients undergoing gastrointestinal endoscopy: a randomized controlled trial
Source: BMC Anesthesiol. 2023 Mar 28;23:93. doi: 10.1186/s12871-023-02053-9 (PMC10044711; doi:10.1186/s12871-023-02053-9)
Supplement: Supplementary file 1 — Additional file 1. [file 12871_2023_2053_MOESM1_ESM.docx]

**Supplemental Table 1** Subgroup of propofol consumption (median and interquartile range)

|  | Group I: Butorphanol  (n=95) | Group II: Normal Saline  (n-99) | Z | Sig |
| --- | --- | --- | --- | --- |
| Colonoscopy | 170(130-180) | 200(150-252) | -2.972 | 0.003 |
| Gastro-colonoscopy | 265(222-315) | 300(250-350) | -1.939 | 0.053 |
| Z | -6.999 | -5.663 |  |  |
| Sig | ＜0.001 | ＜0.001 |  |  |

**Supplemental Table 2** Subgroup of operative time (median and interquartile range)

|  | Group I: Butorphanol  (n=95) | Group II: Normal Saline  (n-99) | Z | Sig |
| --- | --- | --- | --- | --- |
| Colonoscopy | 15(10-20) | 15(10-25) | -1.889 | 0.059 |
| Gastro-colonoscopy | 18(14-28) | 17(12-26) | -0.430 | 0.667 |
| Z | -3.095 | -0.777 |  |  |
| Sig | 0.002 | 0.437 |  |  |
